# 2D and 3D Immobilization of Carbon Nanomaterials into PEDOT via Electropolymerization of a Functional Bis-EDOT Monomer

**DOI:** 10.3390/polym13030436

**Published:** 2021-01-29

**Authors:** Antonio Dominguez-Alfaro, I. Jénnifer Gómez, Nuria Alegret, David Mecerreyes, Maurizio Prato

**Affiliations:** 1 CIC biomaGUNE, Basque Research and Technology Alliance (BRTA), 20014 Donostia-San Sebastián, Spain; antoniodominguezalfaro@gmail.com (A.D.-A.); gomez.perez@ceitec.muni.cz (I.J.G.); david.mecerreyes@ehu.es (D.M.); prato@units.it (M.P.); 2 POLYMAT, University of the Basque Country, UPV/EHU, 20018 Donostia-San Sebastián, Spain; 3 Department of Chemical and Pharmaceutical Sciences, University of Trieste, 34127 Trieste, Italy; 4 Basque Foundation for Science, Ikerbasque, 48013 Bilbao, Spain

**Keywords:** fullerenes, CNT, PEDOT, electropolymerization, functional EDOT monomers

## Abstract

Carbon nanomaterials (CNMs) and conjugated polymers (CPs) are actively investigated in applications such as optics, catalysis, solar cells, and tissue engineering. Generally, CNMs are implemented in devices where the relationship between the active elements and the micro and nanostructure has a crucial role. However, they present some limitations related to solubility, processibility and release or degradability that affect their manufacturing. CPs, such as poly(3,4-ethylenedioxythiophene) (PEDOT) or derivatives can hide this limitation by electrodeposition onto an electrode. In this work we have explored two different CNMs immobilization methods in 2D and 3D structures. First, CNM/CP hybrid 2D films with enhanced electrochemical properties have been developed using bis-malonyl PEDOT and fullerene C_60_. The resulting 2D films nanoparticulate present novel electrochromic properties. Secondly, 3D porous self-standing scaffolds were prepared, containing carbon nanotubes and PEDOT by using the same bis-EDOT co-monomer, which show porosity and topography dependence on the composition. This article shows the validity of electropolymerization to obtain 2D and 3D materials including different carbon nanomaterials and conductive polymers.

## 1. Introduction

The performance of (opto) electronic devices depend not only on the combination of different materials, but also on the film morphology and 2D/3D architecture [1]. For example, in organic solar cell fabrication the active layers are formed by an electron donor blended with an electron accepting material, forming interpenetrating bulk heterojunctions. Therefore, micro- and nano-structured networks can increase the interface area between the donor and the acceptor, enhancing the exciton dissociation process, improving the energy conversion and efficiency of the device [2,3]. Hence, morphology and interface engineering, throughout development and integration of interlayer materials, has a crucial role in the overall operation and optimization of the electronic devices.

Among the conjugated polymers (CPs), the commercially available, poly(3,4-ethylenedioxythiophene) (PEDOT) coupled with polystyrene sulfonate (PSS), is the most commonly-used polymer blend in electronic applications as they possess high conductivity, high transparency in thin films, low oxidation potential and thermal stability [4,5,6,7,8,9]. However, not only commercial PEDOT:PSS has a role in this field, but also derivatives can be used to include functional groups, improving structural properties at the micro- and nano-scale. For example, charged cationic PEDOT has shown excellent results in water-based solar cells exceeding maximum efficiency up to 7% due to inhibition of the repulsion between the anions of the redox couples [10], forming layer-by-layer films throughout ionic interactions or improving the adhesion to an ITO substrate, due to aldehyde presence [11,12]. Furthermore, PEDOT-based devices exhibit significant electrochromic properties, and, more importantly, flexibility in polymeric electrochromic devices (ECD) has become highly demanded for transmittance or reflectance optical applications, such as displays, windows or organic light-emitting diodes (OLEDs), due to their conformability and light weight [13,14]. Even though low-cost and low waste approaches to fabricate flexible ECDs are still yet to be obtained, these types of devices are considered to be the next generation of electronics. Moreover, bi-functional monomers with two thiophene active groups can impact properties such as color [15], conductivity, ion selectivity, and mechanical properties [16,17,18]. In this line, it has been reported the synthesis of the bis-malonyl EDOT that can be covalently attached to C_60_ by cyclopropanation reaction and electrochemically deposited on an electrode, to produce novel ambipolar systems with new properties [19,20].

Beyond the traditional approaches to produce nanostructured and multi-component materials, electropolymerization is ideal in the case of CPs. This electrodeposition method yields CPs with excellent properties, such as high conductivity, homogeneity, and possibility of using multiple monomers [21,22].

On the other side, nano- and micro-structure can be modulated by inclusion of carbon nanomaterials (CNMs) such as fullerenes or carbon nanotubes. Among the common electron-acceptors, C_60_ and its derivatives have been considered as one of the most interesting nanomaterials, due to their excellent electronic properties, transparency, nanoscale structure, lightweight, high electron affinity and enhancement of the photoelectron charge transfer [23]. Fullerenes can accommodate up to six electrons in their delocalized p orbitals, and possess considerable electron mobility in condensed phase arrangements [20]. Therefore, it has been widely used in electronic applications and acceptors for organic photovoltaic devices (OPV) [24,25,26,27,28]. Analogously, carbon nanotubes (CNTs) are widely explored, due to their excellent electronic mobility [29], coupled with a high surface area, ultralight weight, electron-rich properties, and excellent chemical and thermal stability [30,31]. In contrast to C_60_, pristine CNTs are not soluble and possess very limited dispersibility. In fact, they can be hybridized introducing functional groups to overcome this limitation [32]. Besides, CNTs can be manipulated to form vertically aligned forests of nanotubes, increasing the active area, thus enhancing the robustness and interconnectivity. Such architectures have been successfully used in different devices from supercapacitors to wearable sensors [33,34].

Beyond the selection of the right carbon nanomaterial, the limitation to form solid self-standing structures can hamper the fabrication of devices. Fortunately, the immobilization of carbon nanomaterials within CPs solves this problem, improves the interconnections and homogeneously distributes the nanomaterial in the donor–acceptor junctions, providing a material with high efficiency. Multiple studies have been focused in obtaining a good distribution of the fullerene within the PEDOT matrix and avoid nanoparticle aggregation [35,36,37,38,39,40]. We have previously manufactured high quality PEDOT/C_60_ films by co-electrodeposition with excellent C_60_ homogeneity and high reproducibility with enhanced donor–acceptor interface, while retaining their bipolar conducting capabilities, i.e., both hole and electron transport [24,41]. As well, we also showed how CNT can be immobilized within a 3D electrode throughout PEDOT polymerization, resulting in a self-standing material with very unique properties as scaffold for tissue engineering and cell differentiation [42,43].

Herein, we report the immobilization and homogeneous micro distribution of two carbon nanomaterials, C_60_ and CNT, through the co-polymerization of a bifunctional EDOT linked by a malonate bridge monomer (bisEDOT). We have first explored the film formation by co-electrodeposition of EDOT and its bifunctional derivative bisEDOT and evaluated the impact of C_60_ on the structure and the electrochemical, spectro-electrochemical and electrochromic properties of the resulting 2D electrode. Using a similar approach, we have studied the immobilization of CNT in a 3D structure through electropolymerization of the same copolymers PEDOT-co-poly(bisEDOT), abbreviated PbisP, within the interstices of a sacrificial porogen template. After the template removal, self-standing porous tridimensional structures were obtained. The impact of the ratio between the co-monomers and the feed of the electropolymerization on the final composition, microstructure morphology and electrochemical properties were investigated in detail.

## 2. Materials and Methods

### 2.1. Chemicals and Instrumentation

3,4-Ethylenedioxythiophene (EDOT; >97%) and hydroxymethyl EDOT (95%) were purchased from Sigma-Aldrich, Madrid, Spain; malonyl chloride was purchased from Acros Organics, Madrid, Spain. Buckminster fullerene (C_60_; >99%) was supplied from Bucky USA, Houston, TX, USA. Carbon nanotubes (CNT, >95%) were purchased from Nanoamor Inc., Katy, TX, USA (stock# 1237YJS: Inner diameter, 5−10 nm; outside diameter, 20−30 nm; length, 0.5−2 μm). Tetrabutylammonium hexafluorophosphate (TBAPF_6_; >98%) was purchased from TCI Europe N.V., Zwijndrecht, Belgium. The solvents were acquired from Carlo Erba Reagents SAS, Sabadell, Spain. All reagents and solvents were used as received with no further purification. Crystal sugar grain commercially purchased.

Electrochemical experiments were performed using a one compartment, three-electrode electrochemical cell driven by an Autolab MSTAT204 Potentiostat/Galvanostat (Methrom, Madrid, Spain). Flexible Indium Tin Oxide (ITO) coated PET slices, purchased from Sigma-Aldrich, Madrid, Spain, were used as both working and counter electrodes with dimensions of 5 mm × 20 mm. Ag/AgCl was used as the reference electrode.

Atomic force microscopy (AFM) images were obtained with a Nanoscope IIIa (VEECO Instruments, Plainview, NY, USA). As a general procedure to perform AFM analyses, tapping mode with a OTESPA-R3 probe (300 kHz; 26 N/m) (Bruker, Billerica, MA, USA) were carried out. The obtained AFM-images were analyzed in Gwyddion 2.46. Scanning electron microscopy (SEM) was performed JEOL JSM-6490LV at 15kV, running in a point by point scanning mode.

Matrix-free LDI-Tof mass analyses were performed on an Ultraflextreme III time-of-flight mass spectrometer equipped with a pulsed Nd:YAG laser (355 nm) and controlled by FlexControl 3.3 software (Bruker Daltonics, Bremen, Germany). The acquisitions (total of 2000–3000) were carried out in positive reflector ion mode with pulse duration of 70 ns, laser fluence of 50% and laser frequency of 1 kHz. Laser intensity was set marginally above the threshold of ionization to avoid fragmentation (less than 20% for all the cases). C_60_ was detected [M]+ with high intensity signal (>5000 a.u.). The m/z range was set between 400 and 1200. The acquired data was processed (baseline substraction and normalized) using the software FlexAnalysis 3.3 (Bruker Daltonics, Bremen, Germany).

X-ray photoelectron spectroscopy (XPS) analysis were performed using a SPECS SAGE HR 100 system equipped with 100 mm mean radius PHOIBOS analyzer. The deconvolution of C 1s, N 1s, S 2p and O 1s signals were evaluated individually using Casa XPS 2.3.17 software. For the fitting, the Shirley type background subtraction was used, and all curves were defined as 30% Lorentzian, 70% Gaussian. Besides, constrains of the full width at a half maximum (FWHM) and the peak positions were applied. Binding energy calibration was referred on graphitic C–C/C=C at 284.42 eV. The measurements were carried out directly onto PEDOT-co-poly(bisPEDOT) (PbisP) or PEDOT-co-poly(bisEDOT)/CNT (PbisP/CNT) scaffolds attached onto the ITO electrode.

Thermogravimetric analyses (TGA) were performed under air (25 mL·min^−1^ flow rate) using a TGA Discovery (TA Instruments, L’Hospitalet de Llobregat, Spain). The samples were equilibrated at 100 °C for 20 min and then heated at a rate of 10 °C·min^−1^, in the range from 100 °C to 800 °C.

### 2.2. Synthesis of BisEDOT Monomer

BisEDOT was synthesized as reported elsewhere [20]. Briefly, hydroxymethyl EDOT (250 mg, 1.45 mmol) and pyridine (146.7 mg, 1.85 mmol) was dissolved in 1 mL of tetrahydrofuran (THF) anhydrous. Malonyl chloride was then dissolved in 1.5 mL of THF and added drop by drop to the solution at 0 °C. The reaction was stirred at room temperature during 24 h, and then the solvent was removed with vacuum. The solid was extracted with dichloromethane, washed with water, ammonium chloride and lastly with a saturated solution of sodium chloride. The organic phase was then dried over Na_2_SO_4_ and the solvent was removed in vacuum to furnish a green oil. Finally, the oily sample was purified through column chromatography on silica gel with CH_2_Cl_2_ as the eluting solvent. The final product was collected as an oil. Yield: 40%. ^1^H NMR (500 MHz, CDCl_3_) δ 6.38 (d, 4H), 4.48–4.36 (m, 6H), 4.29–4.19 (m, 2H), 4.13–4.03 (m, 2H), 3.53 (s, 2H). ESI-MS [m/z] = 412.0366 [M + H] ^+^ (412.0287 calculated for C_17_H_16_O_8_S_2_).

### 2.3. C_60_-Based 2D Electrodes Fabrication

CPs/C_60_ were electrochemically synthesized via electrochemical polymerization on flexible ITO electrodes (area of deposition of 25 mm^2^), being C_60_ the doping agent. In order to determine the influence of the fullerene, we have also performed the analogous experiments without C_60_, assuming that part of the salt used as supporting electrolyte, i.e., PF_6_, will act as the doping agent as well forming CPs/PF_6_ films. A total of four different films have been performed: (i) Poly(bisEDOT)/C_60_ (bisP/C_60_); (ii) poly(bisPEDOT) (bisP) (iii) PbisP/C_60_ and (iv) PbisP. The monomer concentration was 50 mM for bisEDOT homopolymer, i.e., experiments (i) and (ii), and 25 mM of each monomer for the copolymerizaton, i.e., experiments (iii) and (iv). The concentration of C_60_ was left constant as 0.5 mM. The deposition was performed running 20 or 100 cycles at 50 mV/s from 0.0 to +1.5 V vs. Ag/AgCl reference electrode. TBAPF_6_ (0.1 M) was used as electrolyte in a 10 mL toluene/acetonitrile solution (4:1, *v/v*). After each deposition process, the resulting film was rinsed with toluene and acetonitrile to remove the unreacted precursors and the electrolyte.

### 2.4. PEDOT-Co-Poly(bisEDOT)/CNT 3D Electrodes Fabrication

**Figure 1:**
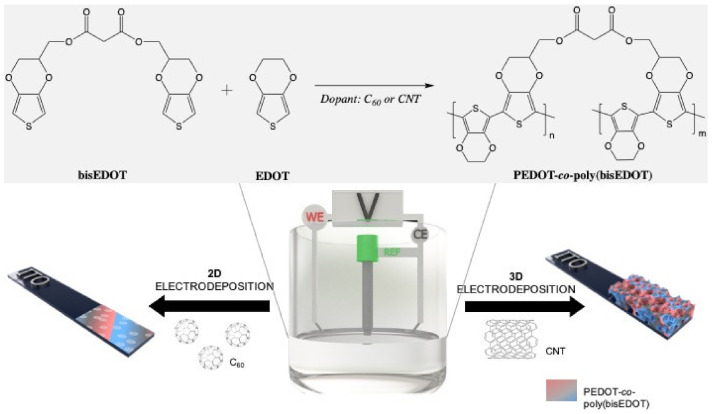
Electrochemical deposition of carbon nanomaterials throughout poly(bisEDOT) and PEDOT-co-poly(bisEDOT) copolymerization within 2D and 3D structures.

Sucrose-based templates were produced through a multi-stage process as previously reported [42]. Briefly, 250 mg of crystal sucrose were smashed and sifted through two sieves with mesh sizes of 100 μm and 250 μm, respectively (Fisher Scientific Inc., Madrid, Spain). Crystal sugar grains in the middle fraction were collected, ensuring a grain size under 250 μm and above 100 μm. CNTs (15 mg) and the sieved sucrose (500 mg) were mixed and shaken overnight. Ten microliters of MilliQ water was added and homogenized before the preparation of the electrodes. The working and counter electrodes were then manufactured on ITO coated PET slides of 5 cm × 30 mm for square electrodes or 20 mm × 20 mm for cylinder electrodes (see Figure 1 for a schematic representation). The co-electropolymerization of EDOT-co-bisEDOT was performed in a three electrodes cell chamber, using Ag/AgCl as the reference and TBAPF_6_ (0.1 M) as supporting electrolyte in acetonitrile (ACN). The solution was purged with Argon before polymerization. The electrodeposition was achieved at a constant potential mode 1.2 V during 2 h and using a total concentration of the monomers of 0.2 M each. Once the polymerization was complete, the scaffold was immersed overnight into MilliQ water to dissolve sucrose, resulting in a self-standing PbisP/CNT architecture with interconnected micro-channels and holes. Control samples without CNT were polymerized in the same conditions, i.e., 1.2 V, 0.2 M, and 2 h.

### 2.5. Cyclic Voltammetry

The cyclic voltammograms of the C_60_-containing films were recorded in the range from −1.0 V to 1.5 V, at a scan rate of 50 mV/s in a 10 mL solution of TBAPF_6_ (0.1 M) in 10 mL toluene/acetonitrile (4:1, *v/v*) solution. As reference, the electrode of Ag/AgCl was used. All measurements were carried out using fresh ITO slices as counter electrodes and fresh solutions at room temperature.

The 3D PbisP/CNT scaffolds were smashed into a powder, dispersed in water, drop-casted on an ITO electrode and dried at 80 °C for 2 h. The amount of material deposited was measured by weight. The area covered with the material was approximately 0.5 cm^2^. CV was then performed in a three-electrode cell, using the drop-casted material as the working electrode, ITO as counter electrode and Ag/AgCl as reference. Cycles were carried out from −0.5 to 1.2 V, using 0.1 M of TBAPF_6_ in acetonitrile (ACN) as the supporting electrolyte.

## 3. Results and Discussion

Carbon nanomaterials (CNMs) were immobilized in the working electrode via an electro-co-deposition within a conjugated polymer (CP). As represented in Figure 1, the electropolymerization between bisEDOT and EDOT was carried out on the working electrode in a three-electrode cell using two different methods, chronoamperometry and cyclic voltammetry. For the fabrication of the 2D films, bisEDOT or EDOT-co-bisEDOT monomers were electropolymerized on the working electrode in presence and in absence of solubilized C_60_ forming a thin film. Instead, for the 3D electrodes, the approach was similar to the one used before [42]: First a sucrose/CNT template was prepared by mixing the two components and molding into specific shape, and was then used as a working electrode during the polymerization and PEDOT-co-poly(bisPEDOT) (PbisP) were co-deposited within such template. Afterwards, the polymerized sucrose/CNT/PbisP structure was immersed into water to remove the sucrose and resulting in a self-standing structure.

### 3.1. 2D Electrodeposition: Immobilization of C_60_ in PEDOT Thin Films 

**Figure 2:**
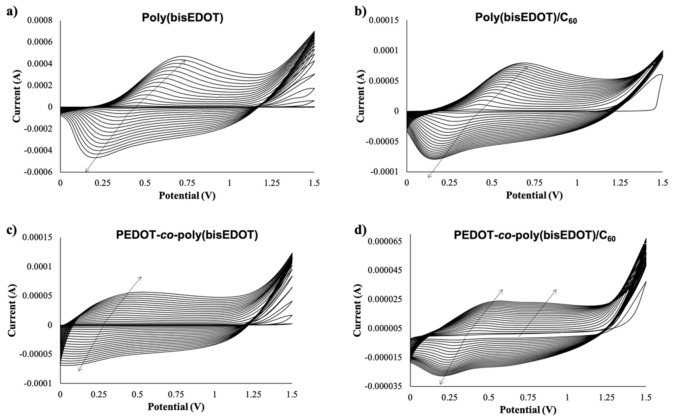
Cyclic voltammetry (20 scans, from 0 V to 1.5 V) for the electropolymerization and deposition of (**a**) bisP (**b**) bisP/C_60_, (**c**) PbisP, and (**d**) PbisP/C_60_.

Due to its electron acceptor properties, electrodeposition of fullerene is feasible at negative values giving rise to poly(fullerene) [24]. Therefore, in order to avoid reduction of the fullerene species, poly(bisEDOT) (bisP) and PbisP were electropolymerized using cyclic voltammetries within the positive range from 0 V to +1.5 V during 20 scans (see Figure 2). Moreover, higher positive potentials may induce over oxidation of the PEDOT-derived compounds. As control, cyclic voltammetry of the polymerization of bisP and co-polymerization of PbisP in the absence of fullerene, i.e., doped with the TBAPF_6_ electrolyte solution, have also been performed. In all the cases, neither anodic nor cathodic peak appeared within the first cycle. As the polymerization progresses, the cycles present an increase of the current and displacement of the band to higher potentials for the oxidation and lower values in the reduction (see arrows in Figure 2), indicating the successful formation of an electroactive film on the ITO electrode surface.

It is well known that PEDOT electropolymerization curves describe a quasi-reversible process with a broad oxidation band corresponding to the transition of the polymer from neutral to oxidized state, and a cathodic counterpart resulting from the inverse transition [24,41]. Interestingly, bisP (Figure 2a) presents remarkable differences, such as the presence of redox peaks with an anodic maximum at 0.6–0.7 V and a cathodic maximum at 0.1–0.2 V. This observation may indicate the formation of coupling products, such as dimers and trimers with lower oxidation potential. Such oligomers are oxidized at lower potentials than the bisEDOT monomer and might tend to form cross-linked networks on the electrode surface. This suggests a limitation in the effective conjugation length, forming smaller particles sizes, which will be corroborated later in this work [44,45]. When the film is formed by PbisP copolymerization (Figure 2c), the curves present an intermediate behavior, i.e., bands at potentials and shapes between those observed for PEDOT and bisP separately, resulting from the combination of both polymerization monomers maintaining the redox properties. In addition, a new band is observed at 0.8 V, which could indicate the formation of larger conjugated particles sizes.

The presence of C_60_ during the different electropolymerizations performed (bisP/C_60_ and PbisP/C_60_) slightly shifts the anodic and cathodic bands to lower potentials, while decreasing remarkably the measured current (see Figure 2b,d). This fact may be due to the early nucleation stage caused by the fullerene that is observed in the current intensities at 1.5 V between the first and second scan. Moreover, the presence of the fullerene as dopant was confirmed by MALDI (see Figure S1).

Is it worth noting that, as seen in previous works, the mechanical stability of the films fabricated is enforced by the presence of the fullerene and no delamination is observed during the manipulation and characterization of the resulting films.

#### 3.1.1. Electrochemical Characterization of the PEDOT/Fullerene Thin Films

**Figure 3:**
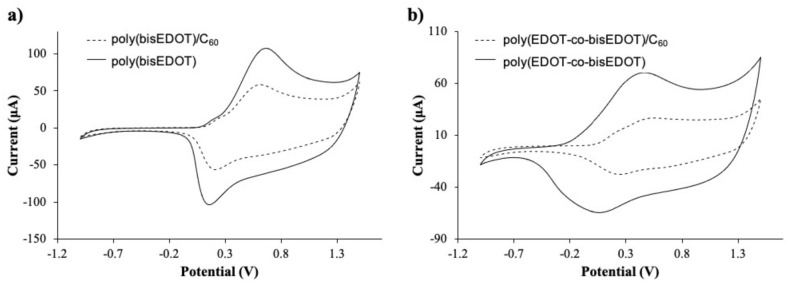
Cyclic voltammograms of the films, 2nd scan of (**a**) bisP and (**b**) PbisP electropolymerized in presence of C_60_ (dotted line) and Tetrabutylammonium hexafluorophosphate (TBAPF_6_) (dark line).

After the electropolymerization, cyclic voltammetry was carried out to confirm the redox activity of the films. Figure 3 and Table 1 presents a comparison between the homopolymer bisP and copolymer PbisP electropolymerized using C_60_ or PF_6_ as doping. The spectra of the homopolymer films containing bisP present an anodic peak above 0.65 V and cathodic peak above 0.20 V. After electropolymerization in presence of C_60_, the anodic peaks are shifted to lower potentials, i.e., 0.60 V while the anodic shifts to higher potentials 0.15 V. As Damlin et al., observed in previous studies, the presence of C_60_ could significantly decrease the redox potentials when multi-layers of polymer are deposited [46]. Therefore, our results confirm the previous hypothesis: bisP forms larger conjugated structures in presence of the fullerene, which produce less redox active nucleus points in the electroactive surface of the electrode, slightly hindering its reduction.

On the other side, copolymerization of EDOT with bisEDOT increases the broad potential, while maintaining the redox activity of the conducting structures. Moreover, the presence of the fullerene can also be confirmed by the reduction in the current on the voltammograms in Figure 3b and Table 1. PbisP shows an anodic band 0.40 V and the corresponding cathodic band above 0.10 V, while in the presence of the fullerene the anodic peak shift to higher potentials (0.45 V) and the cathodic band to 0.25 V, the same tendency as previously observed with the homopolymer. In the same way than the previous observation, we can assume that copolymerization in absence of fullerene produces a larger number of conducting layers than copolymerization in the presence of C_60_.

#### 3.1.2. Electrochromic Analyses

The electrochromic behavior of PEDOT-based films is well understood when doped with conventional anions, such as polystyrene sulfonate (PSS), perchlorate (ClO^4−^), or hexafluorophosphate (PF_6_^−^). The polymer usually suffers a color change depending on its oxidation state: The dark blue of the neutral reduced state (−1.0 V) becomes light blue when the film is fully oxidized (+1.0 V) [47]. We have previously studied the electrochomic response of PEDOT films doped with fullerenes for the first time, and observed a slight color difference, being PEDOT/C_60_ films dark violet in the reduced state. Intermediate shades of blue are observed in the stepwise oxidation corresponding to an optical absorption in the same Visible wavelength range, although with a lower intensity. Furthermore, regarding the PEDOT-derivative bisP used in this work, it is known that polymers with branched substituents have higher bandgaps and the color changes may occur over a smaller voltage range in comparison to the polymers with linear substituents. This is supposed to be due to a limited effective conjugation length allowed in the solid state when branched substituents are present [48]. Therefore, we suggest that the blueshift observed of bisP-based films (480 nm) compared to PEDOT (600 nm) can be explained by the difference of the bandgaps resulting from the branched malonate chain, which can also increase in the spacing between the polymeric chains due to the steric hindrance with the malonate linker [13].

**Figure 4:**
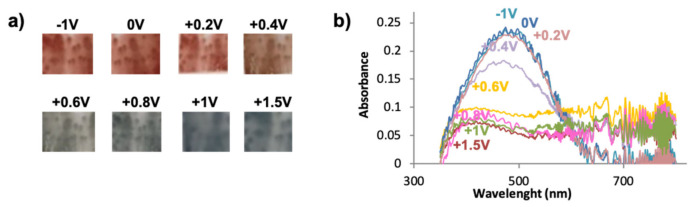
(**a**) Multichromic behavior of bisP/C_60_ at different potentials, (**b**) UV-VIS spectroelectrochemistry for bisP/C_60_.

According to previous observations, we suggest that bisP/C_60_ presents higher crosslinked structures that may have an impact on the electrochromic properties, among others. In order to analyze this aspect, the film was deposited on an ITO electrode and UV-Vis spectra were recorded. At first glance, a color difference is observed with the naked eye in the reduced state of the polymers (−1.0 V, 0.0 V, +0.2 V, and +0.4 V): As shown in Figure 4a, the bisP/C_60_ films are red-brown while, in agreement to the literature, an intense dark violet coloration is observed for PEDOT/C_60_ [41]. Interestingly, light blue coloration appeared when both films were fully oxidized at +1.0 V and +1.5 V, with no evident color difference between the bisP/C_60_ and the PEDOT/C_60_ films.

Figure 4b shows the different absorption spectra of bisP/C_60_ films recorded in the range between 350 nm and 800 nm at various voltages; no further wavelengths were possible to record due to the limitations of the equipment. In good agreement with the literature, the reduced states of PEDOT/C_60_ films present a maximum wavelength of 575 nm (at 0 V) that is displaced towards during the stepwise oxidation, being 600 nm for −1 V and 615 nm for −2 V [41]. Such behavior was new and different from the one observed for the typical PEDOT films, suggesting that the fullerene effect in the coloration is more intense as the negative potential increases. Interestingly, a similar conduct is observed for the bisP/C_60_ films, with a shift in the maximum wavelength for the reduced states, thus confirming the abovementioned change in the color: For voltages from −1 V to +0.2 V, the maximum appeared at 485, while for +0.4 V it was displaced at 475 nm. It is clearly seen that when the films start to get oxidized (+0.6 V), the maximum wavelength band at ~460 nm is bleached. At more positive potentials the 460 nm band is completely bleached, reaching its maximum value when the film is fully oxidized (1.50 V). Similar values are observed for the bisP with no fullerene doping (see Figure S2), suggesting that the effect of the fullerene doping is not as significant as in the PEDOT/C_60_ films. Furthermore, the bands are narrower for the bisP films, with and without doping of C_60_, than those composed of PEDOT. Overall, such observation indicates the completely different behavior and electrochromic response of the bisP-based films in contrast to the underived PEDOT ones.

PEDOT’s six-membered planar ring in comparison with PProDOT’s nonplanar seven-membered ring, resulting in increased spacing along the polymer backbone, which minimizes the stacking of the polymers, and thus decreases electron chain hopping. Therefore, absorption in the near infrared (NIR) is reduced along with the tail into the visible region, making them more transmissive in their oxidized state. By modifying the R groups on P(ProDOT), the color transitions can be tuned across the visible spectrum. The color variability of ProDOTs where two monomers, when copolymerized, can achieve the entire single-wavelength spectrum, but more importantly went to the same clear transparent color when oxidized. An earlier study demonstrated the electrochemical copolymerization of EDOT and thieno[3, 4-b]thiophene (T34bT), and resulted in a red shift of the EDOT λ_max_ [13].

#### 3.1.3. 2D Topography of the BisP/C_60_ Films

**Figure 5:**
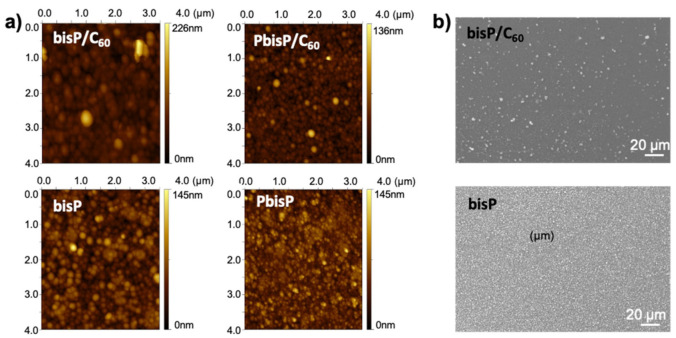
(**a**) AFM images and (**b**) SEM images of electropolymed films onto indium tin oxide (ITO) surface.

In order to confirm all the previous observations, atomic force microscopy (AFM) and scanning electron microscopy (SEM) were performed to analyze the micro- and nano-structure. We evaluated the morphological differences at the nanoscale and microscale. As shown in Figure 5a, the AFM image revealed that the polymer electrodeposited formed a uniform dense layer of round-shaped particles with a grain-like structure. In presence of fullerene, i.e., bisP/C_60_, revealed a uniform layer with particles of around 55 nm in diameter and film thickness up to 90 nm. In absence of the fullerene, the homopolymer bisP presented a relatively increase in the particle size (ca. 60 nm) and a decrease in the film thickness (around 70 nm) (see Figure 5a). On the other hand, the morphology of the PbisP/C_60_ layer produced the smoothest and most homogenous film. The morphology of such PbisP/C_60_ film showed a definite nanoparticle-like grain with about 45 nm in diameter and a thickness of 130 nm. Finally, PbisP film showed a thickness film of 70 nm and 90 nm in particle size (diameter). Overall, the presence of the fullerene as dopant increases the film thickness and produces smaller particles.

As we showed above, bisP and PbisP produce the higher crosslinked structures due to the presence of two thiophene active sides in the monomer, when compared to the previously published PEDOT films [41]. SEM images were performed to corroborate such observations and evaluate at the microscale domain the homopolymer morphology and the effect of the fullerene in the electrodeposition. As clearly observed in Figure 5b, the presence of fullerene increases the average particle size and decreases the particle density. When the electrodeposition is carried out in absence of the fullerene, the particle size decreases and the resulting films show higher homogeneity and particle density. As we observed previously [41], such observation confirms that the fullerene acts as a nucleation point during the polymerization and forms dense areas with higher particle size.

### 3.2. 3D Electrodeposition: Immobilization of Carbon Nanotubes in PEDOT Porous Scaffolds

In contrast with fullerenes, CNT are not soluble in organic solvents. This fact can be advantageous for constructing 3D electrodes: A tridimensional template of crystal sugar grain mixed with CNT can be used as the working electrode during electropolymerization, as reported before [42]. PbisP and PbisP/CNT were obtained using this same approach. Briefly, the electrochemical polymerization of different monomer ratios EDOT-bisEDOT was carried out throughout chronoamperometry at 1.2 V during 2 h and the corresponding polymers were deposited within the interstices of the sugar/CNT template. We have modulated the ratio of both monomers in order to obtain different copolymers and evaluate its impact on the final tridimensional porous structure. Three different amounts of EDOT:bisEDOT ratios (70:30, 95:5, and 97:3 wt.%) were added in the electrochemical cell before starting the reaction. The chronoamperometries show positive curves for all the compositions, indicating the successful oxidation process (Figure S3). The value of the current obtained at the end of the reaction is correlated with the polymer electrodeposited inside the interstices.

As previously demonstrated, the presence of the CNT impacts the growth of the conducting polymer, forming nucleation points in the tridimensional axis moving away from the electrode surface, therefore producing large structures and forming 3D electrodes [42]. After the reaction, the sugar was removed using water, forming a self-standing 3D porous structure. Moreover, porous scaffolds in absence of CNT were also synthesized for comparison. Moreover, the resulting scaffolds with the highest initial amount of EDOT:bisEDOT, i.e., 70:30 wt.%, were not able to maintain the proper 3D structure homogeneously after the sucrose removal and presented a structural morphology partially similar to control samples described below. Furthermore, the scaffolds able to keep the 3D structure, those with the higher initial amount of EDOT (ratios of 95:5 and 97:3 wt.%), resulted in the higher thickness when compare to the control scaffolds without the presence of CNT. Finally, CNM are well-known to enhance the mechanical properties of the composites, and herein the CNT-containing scaffolds obtained showed higher mechanical stability when compared to the CNT-free ones.

#### 3.2.1. Chemical and Electrochemical Characterization of the Thin Films

**Figure 6:**
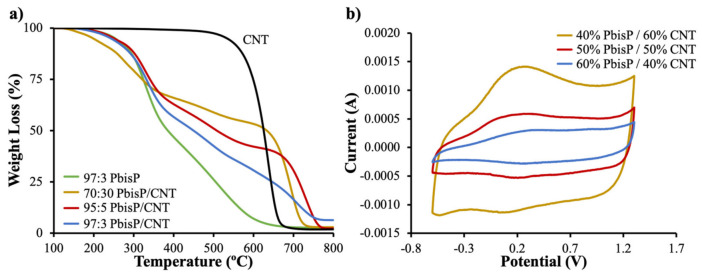
(**a**) Thermogravimetric analyses (TGA) of the different copolymers obtained by electropolymerization and its comparative with control samples (**b**) CVs of the materials carried out at 50 mV/s in acetonitrile and TBAPF_6_.

TGA was used to calculate the polymer amount with respect to CNT for each scaffold (Figure 6a). CNT degradation curve was estimated at a temperature range from 600 to 800 °C, while the scaffold of PbisP synthesized in absence of CNT degrades before 600 °C; see Figure S4 for a detailed plot of the first derivative. Therefore, the polymer percentage of each scaffold was calculated at 500 °C. As a result, scaffolds with 40%, 50%, and 60% (wt.%) of PbisP were obtained for the monomer ratios 70:30, 95:5, and 97:3 (wt.%) respectively. It is remarkable that the polymer deposited within the lattices is increased with the amount of EDOT used. We hypothesize that this observation is due to the lower oxidation potential of bisEDOT, which forms particles-like structures and, therefore, may react with itself, thus inhibiting part of the growth throughout thiophene chains along the tridimensional structure.

In order to confirm the scaffold’s composition in regard of the surface composition of the self-standing scaffolds, XPS analyses were carried out (see Figure S5). All samples present the expected C, O and S peaks beside as F and P corresponding to the doping. The elemental scan of C 1s spectra deconvolution shows the different components within the final polymer scaffold [29,49]. As expected, the electropolymerization with initial 97:3 wt.% of EDOT:bisEDOT produces higher PEDOT polymer than 95:5 wt.% EDOT:bisEDOT, as can be confirmed by the decrease in the oxygen atomic percentage from 23% to 17% that indicates the decrease of the malonate species. Moving to a deeper analysis of the high-resolution deconvolution C peak, there is an increase of the intensity related to C–C/C=C bond (284.5 eV), being of 30% for the scaffolds with higher amount of PEDOT, i.e., PbisP/CNT of 60% and 50% of polymer composition according to the TGA, compared to the 24% of the CNT-free scaffold PbisP. As well, the π-π* band (290.9 eV), which is not present in the PbisP scaffolds, is clearly visible in the CNT-containing sample. All these facts confirmed the presence of CNTs on the scaffolds surface.

The presence of the bifunctional thiophene bisP was confirmed using CVs. As shown in Figure 6b, the PbisP/CNT scaffold with the highest amount of PEDOT, i.e., 40% of bisEDOT according to TGA, has an anodic band above 0.2 V with a cathodic sharp band at 0.1 V. The increment of the bifunctional component, i.e., 50% and 60% of polymer according to TGA, resulted in a dramatic decrease of the current, in addition to slight changes to lower potentials in the case of the anodic band (0.3 V) and higher potential cathodic band (0.35 V).

#### 3.2.2. Morphology of the 3D Structures

**Figure 7:**
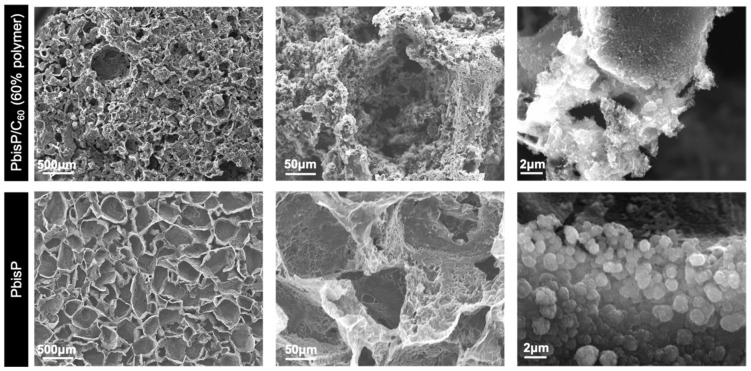
SEM images of the PbisP/CNT scaffolds with 60% of polymer content (top) and PbisP (bottom) after sucrose removal. Synthesis conditions: 1.2 V, 0.2 M of bisEDOT, and 2 h of electrodeposition.

As previously pointed out, the PbisP/CNT scaffolds with 50% and 60% of polymer composition according to TGA, produced a tridimensional structure with different thickness after the cleaning step. In order to analyze the impact of the polymer and CNT in the microscale, SEM was performed (see Figure 7 and Figure S6). Besides, control samples synthesized at the same conditions in CNT-free templates were also analyzed (see Figure S7). PbisP/CNT with 60% of polymer content resulted in the larger structure with a thickness of 429 µm, while its homologous PbisP without CNT was around 20% thinner. As well, the structure of PbisP/CNT with 50% of polymer content, presented a height of 363 µm that decreases among the half when it was electropolymerized in the absence of CNT. Moreover, the composition not only impacts on the thickness profile, but also modulates the surface morphology: While PbisP/CNT scaffolds tend to remain in the tridimensional structure and create interconnected pores, PbisP architectures result in an opened-porous structure with absence of interconnections. Moreover, the presence of brush-like structures at higher magnifications can be observed in the presence of CNT scaffolds, while particles are present in majority in the PbisP structures, in agreement with the results obtained in the 2D electropolymerization.

## 4. Conclusions

In this work, we have explored a co-electrodeposition method to immobilize fullerene C_60_ and CNT in 2D and 3D conductive polymer PEDOT, respectively. For this purpose, we have synthesized a bi-functional EDOT derivative and investigated its co-electropolymerization with EDOT.

First, 2D thin films have been manufactured using cyclic voltammetry in the presence and in absence of fullerene C_60_, producing copolymer PbisP and homopolymer bisP doped with and without C_60_. Then, the impact of the fullerene doping and the crosslinked structure has been studied by CVs and spectroelectrochemical methods. By modulating the monomers ratios, we have changed the morphology observed at the micro- and nano- domain by AFM and SEM, producing electroactive films with different particles size and particles density.

Second, we have prepared conductive porous scaffolds by a copolymerization of PbisP onto sucrose/CNT 3D electrode templates. The relative composition of the scaffolds was modulated between 40 and 60 wt.% PbisP respect to CNTs. The copolymer composition has been corroborated by CVs and XPS. Moreover, using this method the microscopic structure of the electrode can be modulated and shown to affect not only the frontal topography but also the thickness of the electrodes.

This article shows the validity of electropolymerization to obtain 2D and 3D materials containing different carbon nanomaterials and conjugated polymers. The application of these new donor/acceptor nanostructures in opto and bioelectronic devices will be investigated in the near future.

## Figures and Tables

**Table 1:** Anodic and cathodic bands corresponding to the bisP homopolymer and PbisP copolymer in absence and presence of fullerene C_60_.

Sample	E_0_/E_+1_	E_+1_/E_0_	E_0_/E_−1_	From
**bisP/C_60_**	0.60 V	0.15 V	–	Figure 3a
**bisP**	0.65 V	0.20 V	–	Figure 3a
**PbisP/C_60_**	0.45 V	0.25 V	–	Figure 3b
**PbisP**	0.40 V	0.10 V	–	Figure 3b
**PEDOT/C_60_**	0.80 V	−0.40 V	−0.85 V	[41]
**PEDOT**	0.55 V	−0.25 V	−0.7 V	[41]

## Supplementary Materials

The Supporting Information is available online at https://www.mdpi.com/2073-4360/13/3/436/s1. Figure S1. MALDI-TOF of the poly(bisEDOT)/C_60_ scaffold showing the presence of the fullerene. Figure S2. UV-VIS spectroelectrochemistry for bisP. Figure S3. Chronoamperometry of the different compositions scaffolds PEDOT-co-POLY(bisEDOT)/CNT compared with poly(bisEDOT) at 1.2V during 2h. Figure S4. First derivative analysis from the TGA plot. Figure S5. a–b) XPS analysis of the different composition scaffolds and its corresponding atomic percentage. c) C1s and its deconvolution of poly(EDOT-co-bisEDOT), 50% poly(EDOT-co-bisEDOT)/CNT and 60% poly(EDOT-co-bisEDOT)/CNT scaffolds. Figure S6. SEM images at different magnifications for 40% PbisP/CNT scaffolds (top) and 50% PbisP/CNT (bottom). Figure S7. SEM images of scaffolds’ profile for different compositions electropolymerized with and without CNT.
