# Supplementary figures and images for: 2D and 3D Immobilization of Carbon Nanomaterials into PEDOT via Electropolymerization of a Functional Bis-EDOT Monomer
Source: Polymers (Basel). 2021 Jan 29;13(3):436. doi: 10.3390/polym13030436 (PMC7866415; doi:10.3390/polym13030436)

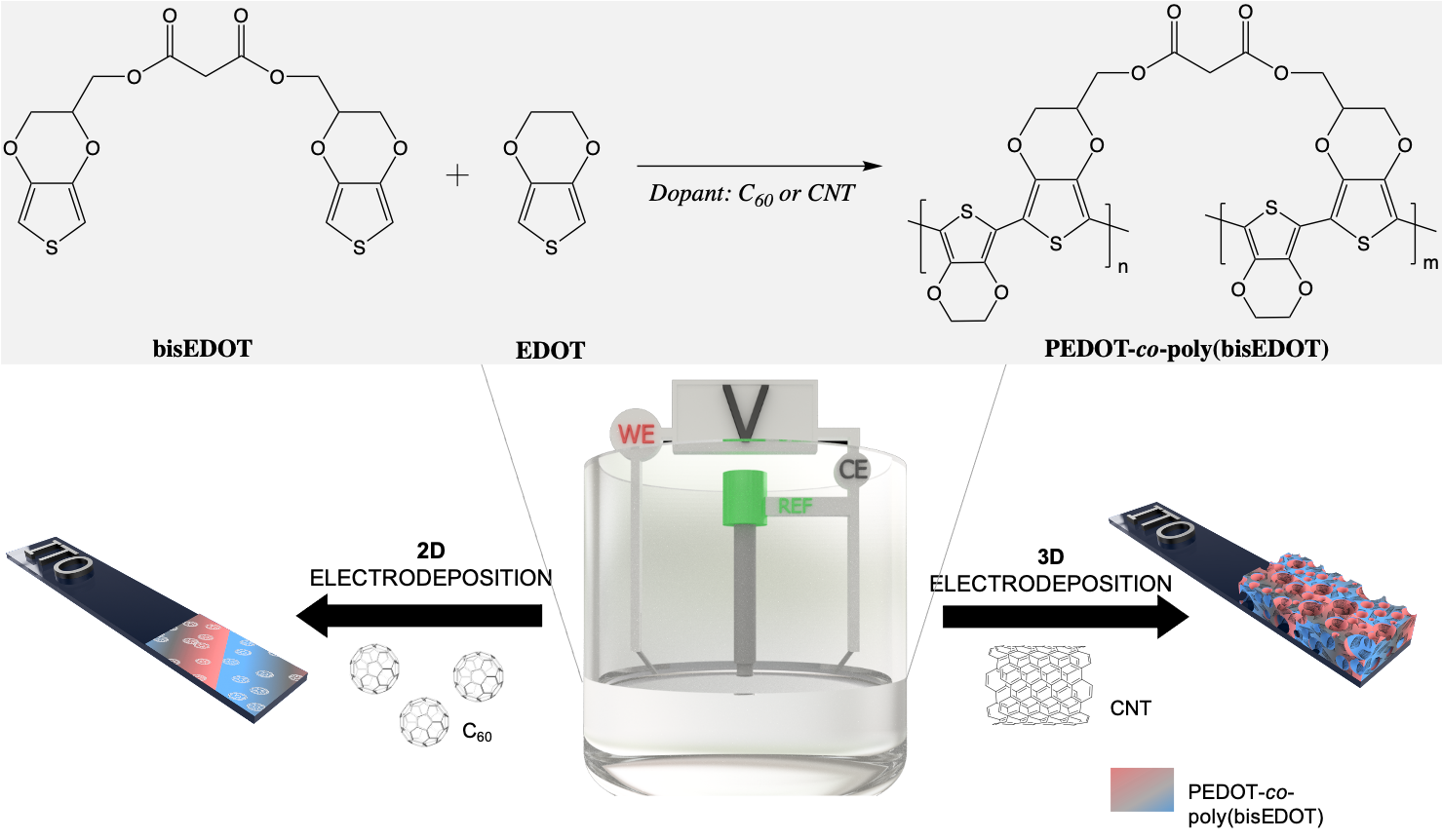

Supplement: Supplementary file 1 [file polymers-13-00436-s001.zip › Fig1.png]

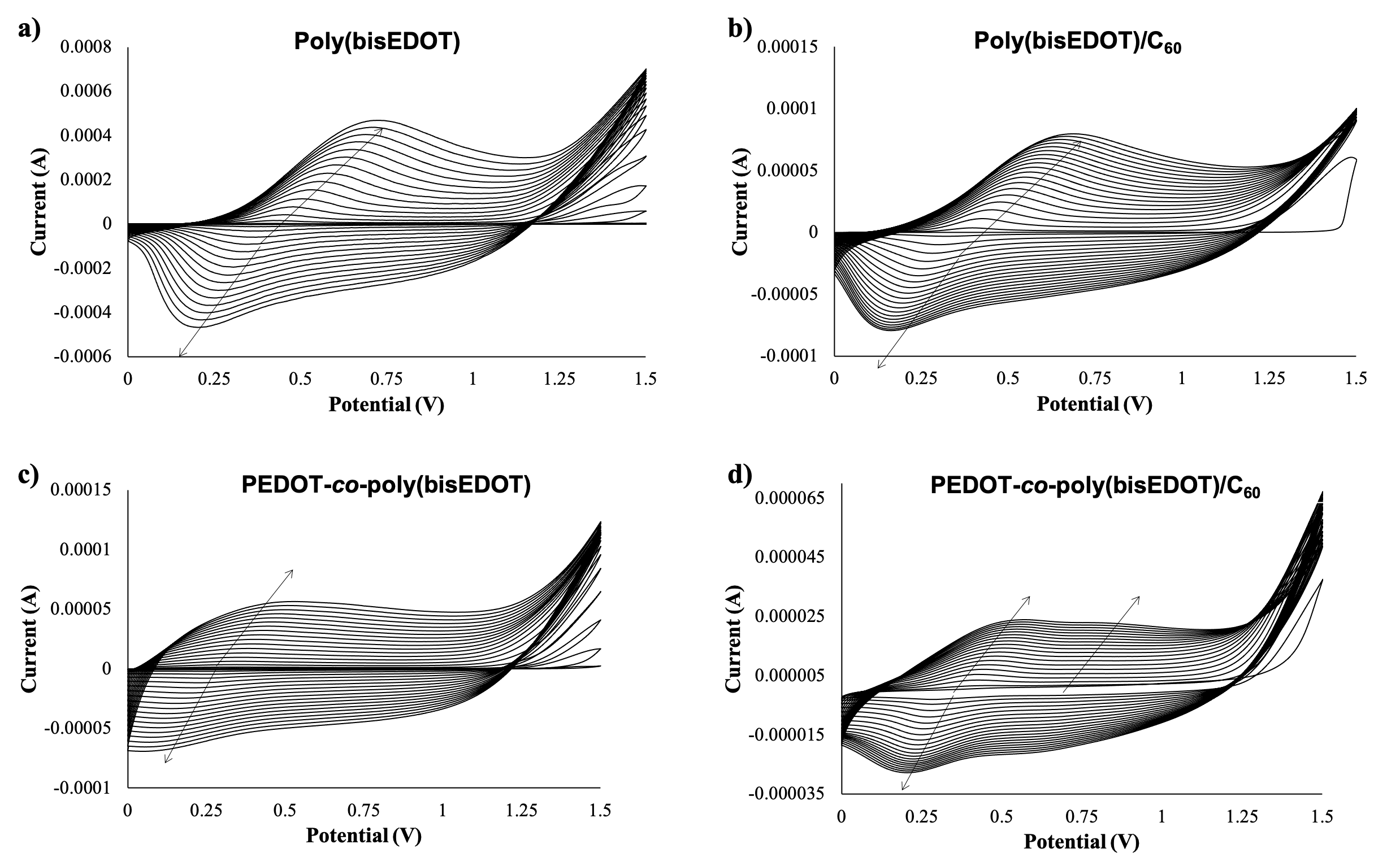

Supplement: Supplementary file 1 [file polymers-13-00436-s001.zip › Fig2.png]

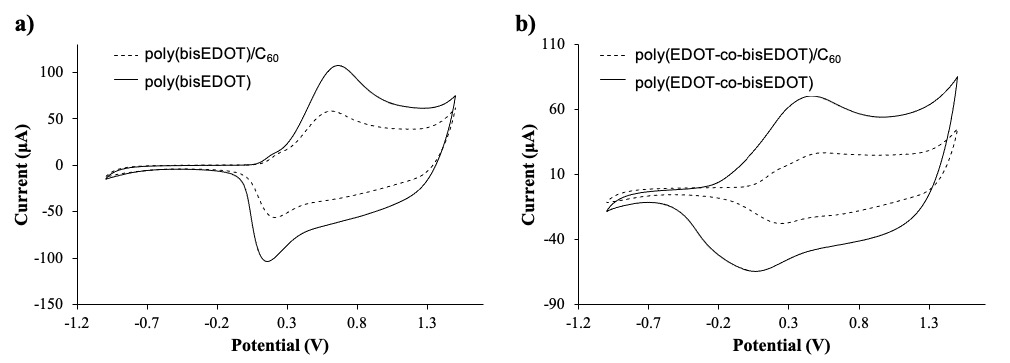

Supplement: Supplementary file 1 [file polymers-13-00436-s001.zip › Fig3.jpg]

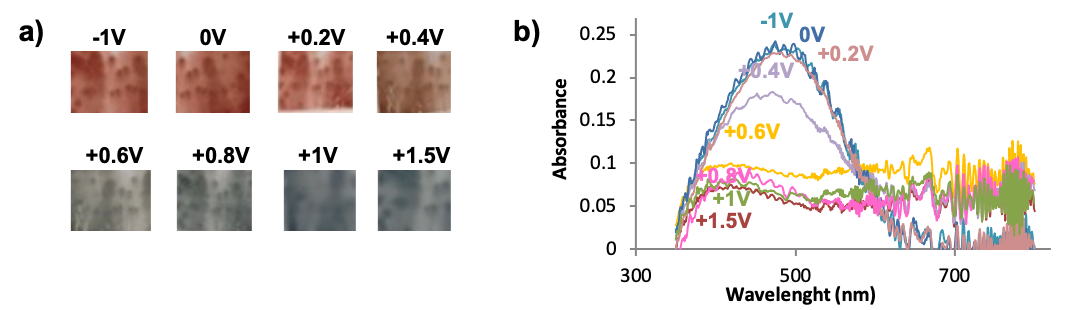

Supplement: Supplementary file 1 [file polymers-13-00436-s001.zip › Fig4.png]

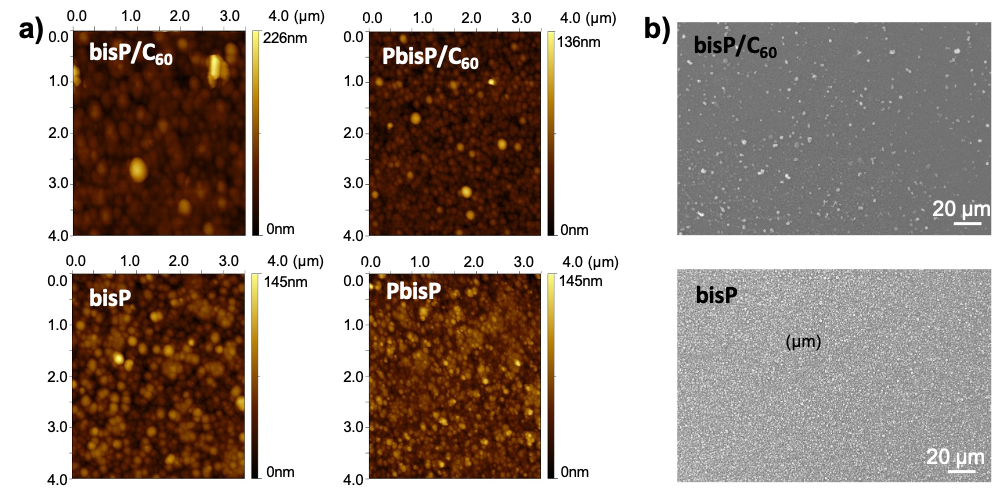

Supplement: Supplementary file 1 [file polymers-13-00436-s001.zip › Fig5.png]

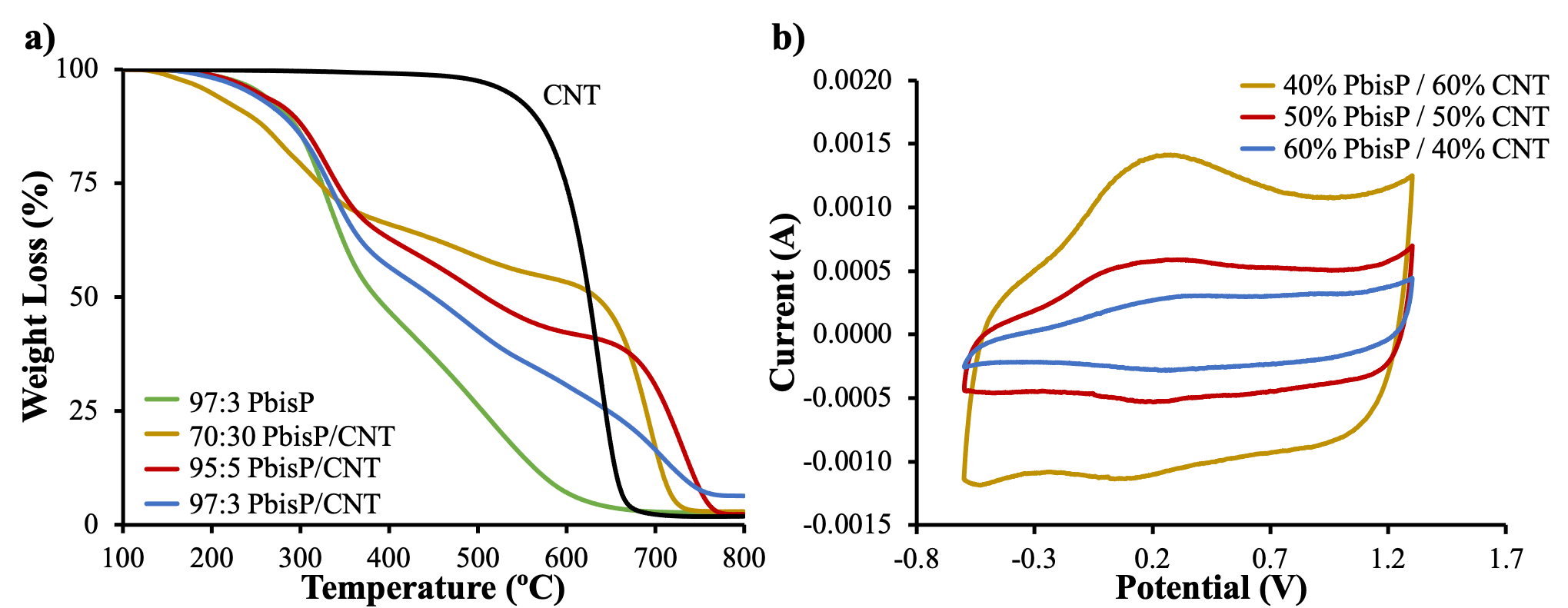

Supplement: Supplementary file 1 [file polymers-13-00436-s001.zip › Fig6.png]

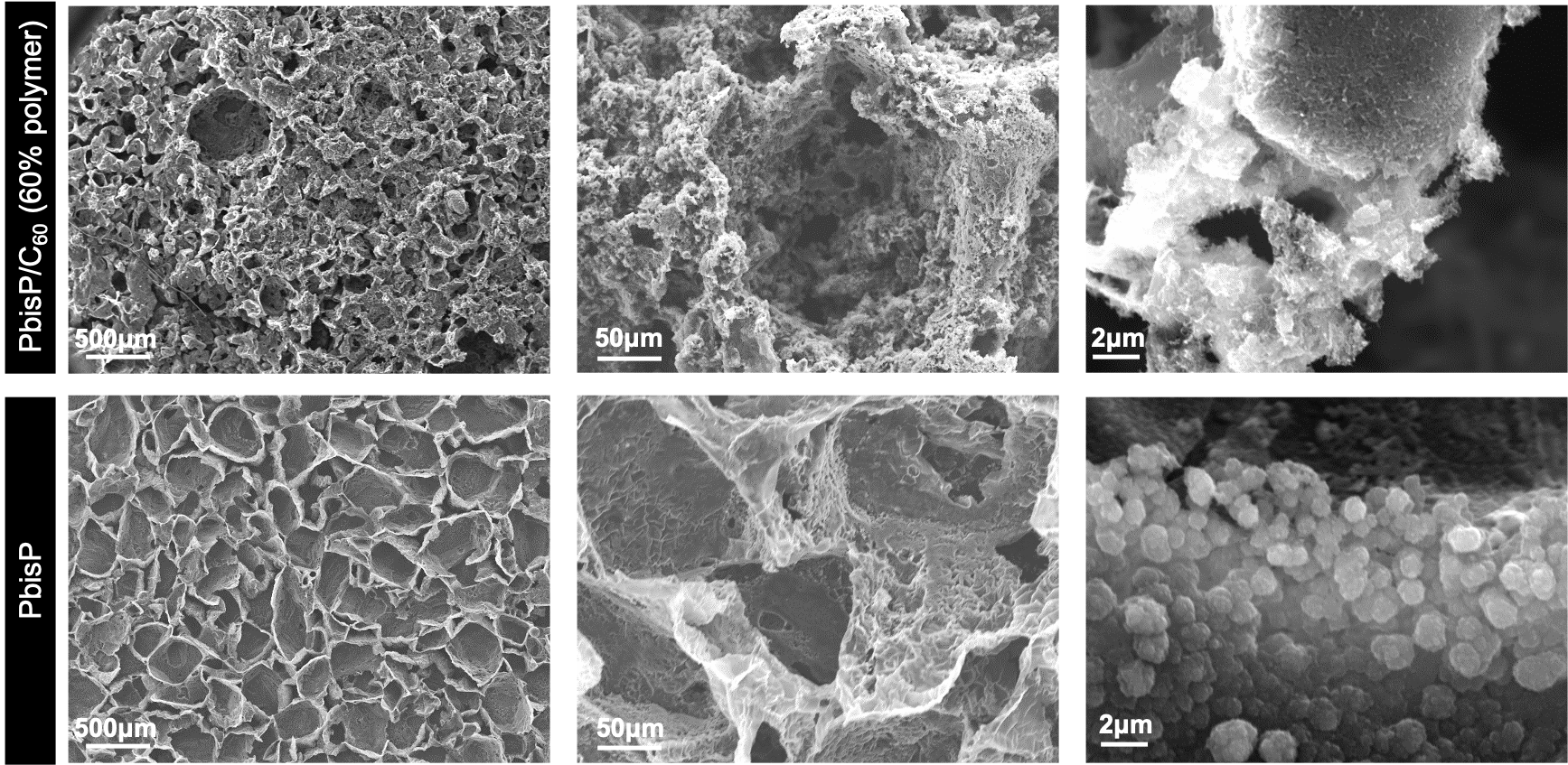

Supplement: Supplementary file 1 [file polymers-13-00436-s001.zip › Fig7.png]

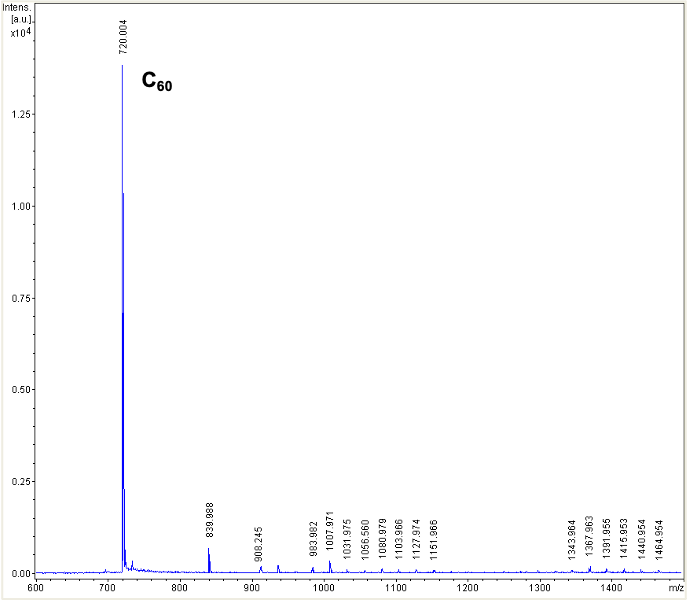

Supplement: Supplementary file 1 [file polymers-13-00436-s001.zip › FigS1.png]

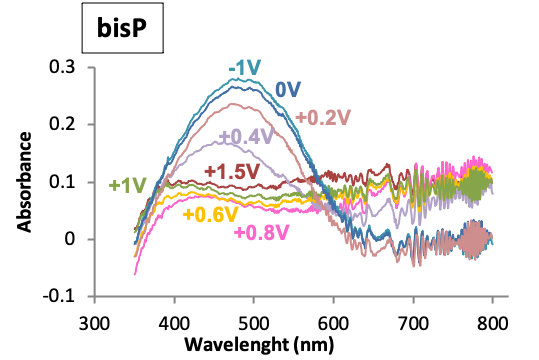

Supplement: Supplementary file 1 [file polymers-13-00436-s001.zip › FigS2.png]

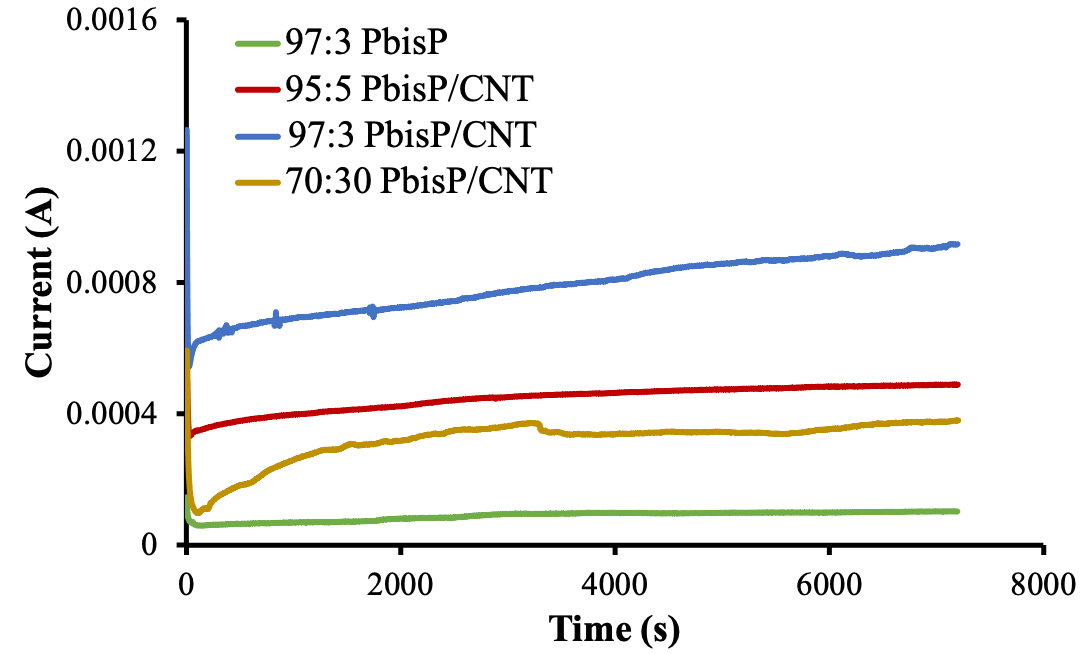

Supplement: Supplementary file 1 [file polymers-13-00436-s001.zip › FigS3.png]

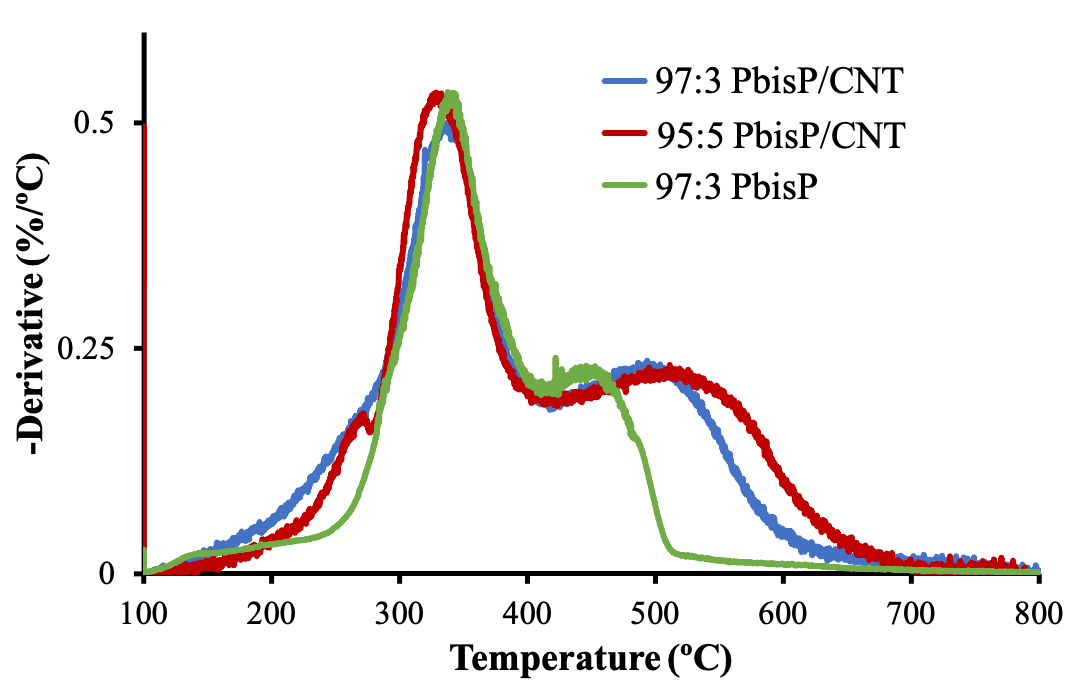

Supplement: Supplementary file 1 [file polymers-13-00436-s001.zip › FigS4.png]

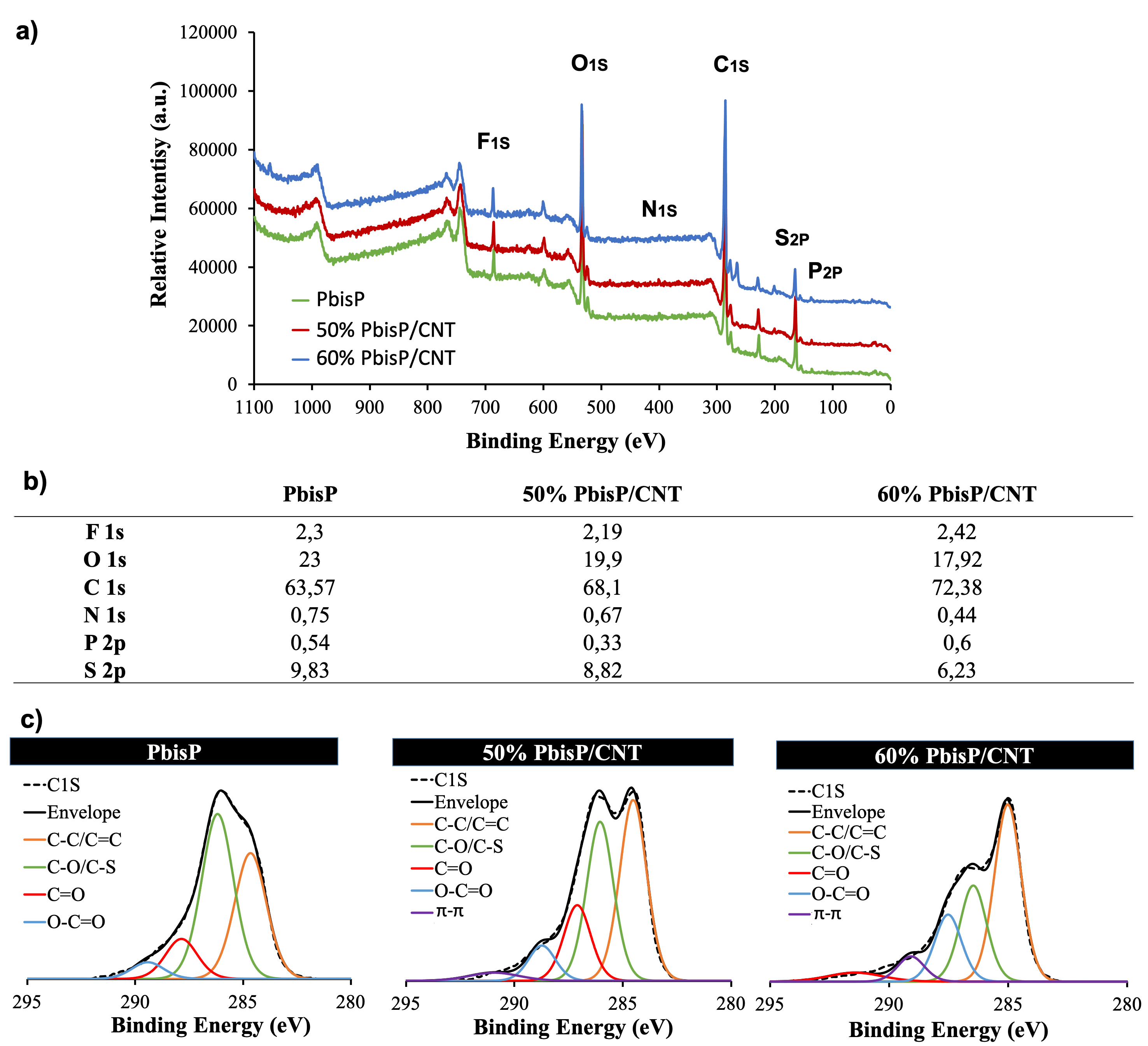

Supplement: Supplementary file 1 [file polymers-13-00436-s001.zip › FigS5.png]

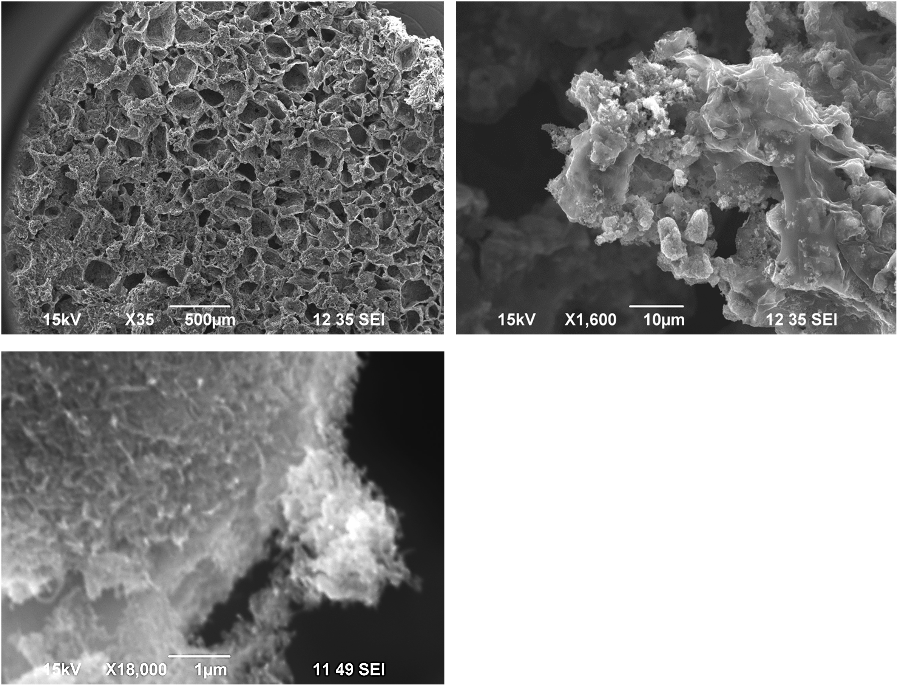

Supplement: Supplementary file 1 [file polymers-13-00436-s001.zip › FigS6.png]

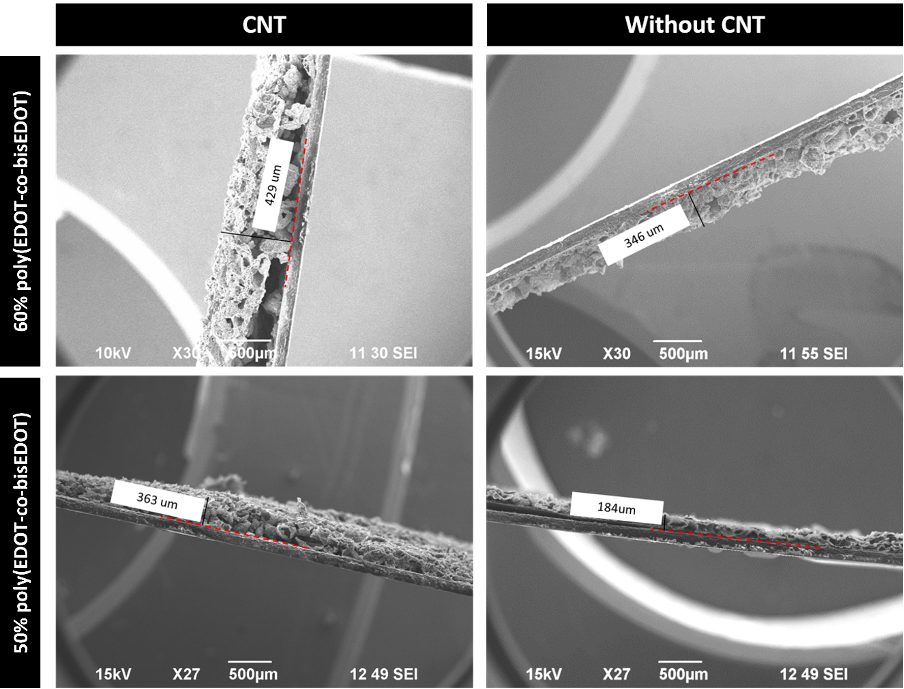

Supplement: Supplementary file 1 [file polymers-13-00436-s001.zip › FigS7.png]
